# Carriage of Capsular Serotype K1 Klebsiella pneumoniae Sequence Type 23 Strains in Healthy Microbiology Laboratory Staff in Russia

**DOI:** 10.1128/MRA.00349-21

**Published:** 2021-05-13

**Authors:** Ekaterina S. Kuzina, Tatiana S. Novikova, Viktor I. Solomentsev, Angelika A. Sizova, Eugeny I. Astashkin, Nadezhda K. Fursova

**Affiliations:** a State Research Center for Applied Microbiology and Biotechnology, Obolensk, Moscow Region, Russia; University of Maryland School of Medicine

## Abstract

Klebsiella pneumoniae causes both nosocomial and community-associated infections. Among the hypervirulent K. pneumoniae (hvKP) isolates, K1 is the most common capsular serotype. Here, we report the draft genome sequences of 3 K1-type (sequence type 23) K. pneumoniae strains isolated from healthy microbiology laboratory staff in Russia.

## ANNOUNCEMENT

Asymptomatically colonizing strains of capsular serotype K1 Klebsiella pneumoniae sequence type 23 (ST23) have been reported as related to liver abscess in South Korea (1, 2) and recently in the United States (3). In Europe, the colonization of healthy volunteers by K. pneumoniae of high clonal diversity genetic lines is not associated with severe infections (4).

In this study, we analyzed the asymptomatic carriage of K. pneumoniae by microbiology laboratory staff (*n* = 33) in the Moscow Region in Russia. This study was approved by agreement of the Federal State Budgetary Educational Institution of Higher Professional Education, A. I. Evdokimov Moscow State University of Medicine and Dentistry (number 11-18, 20 December 2018).

**FIG 1:**
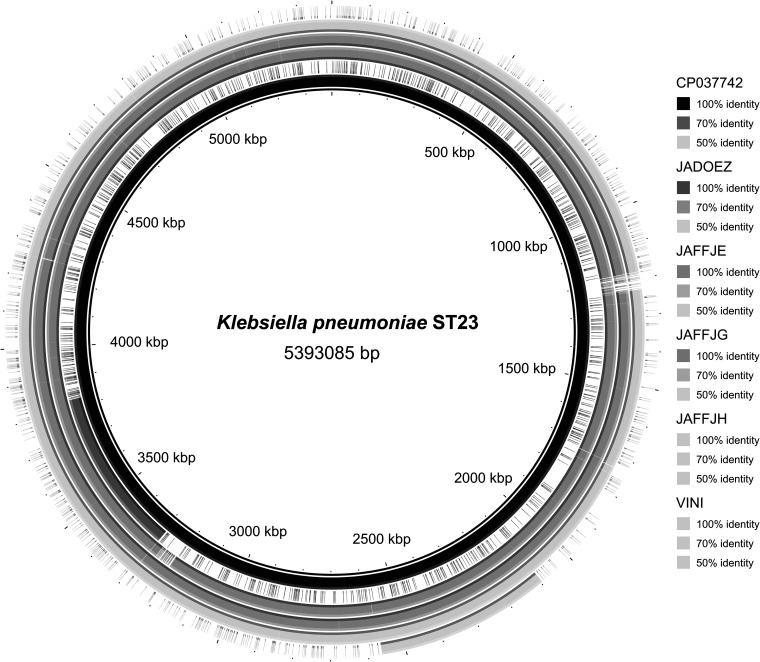
Whole-genome comparisons of the K. pneumoniae strain whole-genome sequences using BRIG v0.95. From the center to the outside: ring 1, reference genome under GenBank accession number CP037742.1 (USA, 2019); ring 2, NZ_JADOEZ000000000.1 (Russia, 2014); ring 3, SCPM-O-B-9260 (JAFFJE000000000) (Z27-Kp/19, Russia, 2019); ring 4, SCPM-O-B-9258 (JAFFJG000000000) (F19R-1Kp/19, Russia, 2019); ring 5, SCPM-O-B-9257 (JAFFJH000000000) (F18R-1Kp/19, Russia, 2019); and ring 6, NZ_VINI01000000.1 (India, 2016).

Gram-negative bacterial isolates (*n* = 100), including those from 87 stool samples and 13 throat samples, were obtained from 33 healthy microbiology laboratory staff. Bacteria were isolated on lactose triphenyl-tetrazolium chloride (TTC) agar with tergitol-7 (SRCAMB, Russia). Identification was done using matrix-assisted laser desorption ionization–time of flight mass spectrometry (MALDI-TOF MS) (Bruker, Germany) with the reference strain K. pneumoniae ATCC 700603. The MICs of antibacterials were determined using a Vitek 2 compact instrument (bioMérieux, France) and interpreted according to the Clinical and Laboratory Standards Institute (CLSI) guidelines (5). Escherichia coli strains ATCC 25922 and ATCC 35218 were used for quality control. The hypermucoviscous phenotype was determined by string test (6). DNA was isolated from overnight cultures grown in Luria-Bertani (LB) broth (Difco, USA) with aeration (120 rpm) at 37°C using a genomic DNA prep kit (BioFact, South Korea). Whole-genome sequencing of K. pneumoniae strains was performed using the Nextera DNA library preparation kit and MiSeq reagent kit v3 (300 cycles) on an Illumina MiSeq platform. The reads without quality filtering were *de novo* assembled using Unicycler v0.4.7 (7) with default parameters (Table 1). The annotation was performed using the NCBI Prokaryotic Genome Annotation Pipeline (8). Multilocus sequence typing (MLST) and identification of antibiotic resistance genes, virulence genes, plasmids, and restriction-modification systems were done using the Web resource of the Center for Genomic Epidemiology (http://www.genomicepidemiology.org/) and the BIGSDB database (https://bigsdb.pasteur.fr/klebsiella/klebsiella.html) (9–11). Whole-genome comparisons were rendered using BRIG v0.95 (12) (Fig. 1).

**TABLE 1:** Strain-identifying information and basic statistics of the assemblies and annotations

Feature	Data for K. pneumoniae strain:		
	F18R-1Kp/19	F19R-1Kp/19	Z27-Kp/19
Sequence type	ST23	ST23	ST23
Capsular type	K1	K1	K1
Isolation source	Stool	Stool	Throat
Collection date	11 April 2019	12 April 2019	12 April 2019
SCPM ID^*a*^	SCPM-O-B-9257	SCPM-O-B-9258	SCPM-O-B-9260
BioSample ID	SAMN17807608	SAMN17807609	SAMN17807611
SRA accession no.	SRR13638374	SRR13638373	SRR13638371
GenBank accession no.	JAFFJH000000000	JAFFJG000000000	JAFFJE000000000
WGS^*b*^ features			
GC content (%)	57.25	57.24	57.25
Genome size (bp)	5,560,322	5,560,734	5,552,064
No. of reads	1,054,272	1,380,544	1,387,698
Mean read depth (×)	251	256	238
Coverage (×)	48.04	65.56	60.02
*N*_50_ (bp)	273,411	208,822	203,441
No. of contigs	74	72	83
No. of genes	5,355	5,361	5,370
Antimicrobial resistance and virulence genes			
Beta-lactam resistance	*bla*_SHV-190_	*bla*_SHV-190_	*bla*_SHV-190_
Fosfomycin resistance	*fosA3*	*fosA3*	*fosA3*
Efflux	*oqxА1*, *oqxB1*	*oqxА1*, *oqxB1*	*oqxА1*, *oqxB1*
Regulator of the mucoid phenotype	*rmpA2*	*rmpA2*
Siderophore salmochelin receptor/synthesis	*iroBCDN*	*iroBCDN*	*iroBCDN*
Klebsiella ferric uptake transporter	*kfuABC*	*kfuABC*	*kfuABC*
Allantoin regulon	*allABCDRS*	*allABCDRS*	*allABCDRS*
Plasmids	IncHI1B, IncFIB	IncHI1B, IncFIB	IncHI1B, IncFIB

a SCPM, State Collection of Pathogenic Microorganisms, Obolensk, Russia; ID, identifier.

b WGS, whole-genome sequencing.

Among 100 Gram-negative isolates, 20 cultures were identified as K. pneumoniae. Three K. pneumoniae isolates collected from 3 persons were characterized as hypermucoviscous bacteria by string test and attributed to sequence type 23 (ST23) and capsular type K1 by whole-genome analysis (Table 1).

The strains were sensitive to cephalosporins, carbapenems, aminoglycosides, ciprofloxacin, and sulfonamides and resistant to ampicillin (MIC ≥ 32 mg/liter) and phosphomycin (MIC ≥ 256 mg/liter). Such phenotypes were associated with the genetic determinants *bla*_SHV-190_, *fosA3*, *oqxA1*, and *oqxB1* (Table 1).

Moreover, the *rmpA* gene, associated with hypermucoviscosity, and the operons *iro*, *kfu*, and *all*, which provide the virulence properties of hypervirulent K. pneumoniae (hvKP), were identified in the genomes (13). Each strain carried two plasmids attributed to the incompatibility groups IncHI1B and IncFIB (Table 1).

This is the first report of hvKP K1 ST23 carriage in healthy people in Russia. Our report shows the need for continued monitoring of hvKP and indicates the importance of clinical awareness of this pathotype.

### Data availability.

The genome sequences were deposited in GenBank under the accession numbers listed in Table 1.
