# Correction for Kuzina et al., “Carriage of Capsular Serotype K1 Klebsiella pneumoniae Sequence Type 23 Strains in Healthy Microbiology Laboratory Staff in Russia”

**DOI:** 10.1128/MRA.00527-21

**Published:** 2021-07-01

**Authors:** Ekaterina S. Kuzina, Tatiana S. Novikova, Viktor I. Solomentsev, Angelika A. Sizova, Eugeny I. Astashkin, Nadezhda V. Kolupaeva, Vasiliy D. Potapov, Nadezhda K. Fursova

**Affiliations:** a State Research Center for Applied Microbiology and Biotechnology, Obolensk, Moscow Region, Russia

## AUTHOR CORRECTION

Volume 10, no. 19, e00349-21, 2021, https://doi.org/10.1128/MRA.00349-21. The article byline should read as given in this correction.

Page 2: In Acknowledgments, “Financial support was provided by a grant from the Ministry of Science and Higher Education of the Russian Federation (number 075-15-2019-1671, 31 October 2019).” should be changed to “The study was supported by the sectoral program of Rospotrebnadzor.”

